# Professional Secrecy and Professional Confidences

**Published:** 1920-07-10

**Authors:** C. O. Hawthorne

**Affiliations:** Harley Street, W. 1.


### THE EDITOR'S LETTER-BOX.

**Correspondence on all subjects is invited, but we cannot in any way be responsible for the opinions expressed by our correspondents, who must give their name and address as a guarantee of good faith, but not necessarily for publication. Correspondents are reminded that brevity of style and conciseness of statement greatly facilitate early insertion.**

### PROFESSIONAL SECRECY AND PROFESSIONAL CONFIDENCES.

*To the Editor of* The Hospital.

Sir,—This topic, at least in one of its aspects, was discussed in The Hospital in June of last year, and I note that it again appears in your issue of the 3rd inst. I found myself on the former occasion in conflict with the editorial views on certain points, and I am glad to note that in the more recent article there is a recognition of the truth that the principle of medical secrecy, as maintained at the meeting of the B.M.A. at Cambridge, rests for its defence on consideration for the public interest. It is not the convenience of the doctors, but the good of the community which justifies the policy of silence. Unless the secrecy of the consulting-room is recognised, sick people, in not a few circumstances, will either not consult doctors at all or will postpone their visits until in desperate straits. That is, the chance of recognising many diseases in their early and curable stages will be diminished and both the individual and the community will be penalised. Here is the defence of the professional habit, and it is seen to rest on a sure and broad foundation.

Some of the comments reported from the B.M.A. meeting show a curious and, as I think, a very narrow and limited view of a medical practitioner's responsibilities. To ask, as Dr. C. Sanders is said to have done, whether the profession ought to allow a "bounder" to live and his wife and child to die may be telling as a rhetorical appeal, but what exactly does it mean? Would Dr. Sanders suggest that the doctor should promptly poison the "bounder?" Or that he should rush to tell the wife and child of their relative's "bounding" achievements? Unless one or other of these steps is taken I do not see what practical advance issues from the exhortation. Nor do I accept Mr. Bishop Harman's contention that a doctor who failed to announce that a given patient is the subject of venereal disease could be held liable for damages should the patient communicate the disease to some other person. Were this argument sound the only protection for the doctor would be to make the announcement by the town crier or public advertisement, or to ask that due intimation be made in the various churches, music-halls, public conveniences, and moving-picture palaces. By no more limited means would it be possible to warn all persons who might possibly be infected, and who might thus, according to Mr. Bishop Harman's legal judgment, be in a position to sue the silent doctor for damages. If the doctor is safe only when he warns of the possible danger everybody who may become involved, publicity on the widest scale is evidently essential. To me it seems that each of these gentlemen is in essential error relative to the medical practitioner's functions in a civilised community. Instead of, or in addition to, being a healer of the sick they wish to make him a judge of morals and the executive officer of an ethical code. Now, whilst I freely allow that a medical practitioner may refuse any person as a patient (though even this ought not to be an arbitrary and unreasoned decision) he is bound, having once accepted the relationship, to do whatever is possible in the patient's interest; and indeed it is for this that he is paid a fee. He may warn the patient of the risk of infecting other persons and may instruct him how such risk may be minimised or removed. But to proceed to make family and public mischief by disclosing what he has learned in confidence is not only a breach of trust, but, in addition, is a prejudice to the common interest by weakening the public faith in the trustworthiness of the profession.

The fact is that the public has means of protection in its own hands but has not the decision or courage to use them; and yet it complains (or some members of it do) because the medical profession will not pull the chestnuts out of the fire. The law might make the deliberate or negligent communication of venereal disease a criminal offence, and, without waiting for the law, it is open to anyone concerned to ask that a marriage contract be conditioned by the provision, by each of the parties to the contract, of a certificate guaranteeing freedom from communicable disease. We are told that public opinion shrinks from steps of this nature. Be it so. But public opinion must accept the consequences of its own timidity and must not try to pass these on to the doctors.

It is no business of the medical profession to estimate either the economic values or the moral merits or demerits of patients; nor, as doctors, do we know anything about bounders on the one hand, or smiling and cherubic innocence on the other. We are concerned with sick men and women who give us their confidence, and of this confidence we strive to be worthy. What verdict on those we endeavour to succour may be passed by political economists or professors of ethics is no concern of ours. To us each is a patient, and it is ours to help him to the utmost of our power. In this fashion, no doubt, we serve the individual. But not less true is it that the practice of loyal and trustworthy medical sympathy and advice is service spent in the interests of the community.—I am, Sir,

Yours faithfully,

Harley Street, W. 1. C. O. Hawthorne.

